# Draft Genome Sequence of *Streptomyces* sp. TP-A0890, a Producer of FR-900452 and A-74863a

**DOI:** 10.1128/genomeA.01212-15

**Published:** 2015-10-15

**Authors:** Hisayuki Komaki, Natsuko Ichikawa, Akira Hosoyama, Nobuyuki Fujita, Yasuhiro Igarashi

**Affiliations:** a Biological Resource Center, National Institute of Technology and Evaluation (NBRC), Chiba, Japan; b NBRC, Tokyo, Japan; c Biotechnology Research Center and Department of Biotechnology, Toyama Prefectural University, Toyama, Japan

## Abstract

Here, we report the draft genome sequence of *Streptomyces* sp. TP-A0890, a producer of FR-900452 and A-74863a. The genome was found to contain at least eight polyketide synthase and nonribosomal peptide synthetase gene clusters. A prediction of gene functions based on the sequence similarity allowed us to assign the biosynthetic gene clusters for FR-900452 and A-74863a.

## GENOME ANNOUNCEMENT

In our continuing investigation of metabolite diversity in actinomycetes, *Streptomyces* sp. TP-A0890, collected from a soil sample, was found to produce a diketopiperazine derivative, FR-900452 (1), and an isoprenoid-polyketide conjugate, A-74863a (2). To date, biosynthetic gene clusters for these compounds have not been reported. In order to evaluate the biosynthetic capacity of this strain and to identify biosynthetic genes for the above-mentioned secondary metabolites, whole-genome shotgun sequencing was conducted.

*Streptomyces* sp. TP-A0890 was deposited at the NBRC culture collection (NBRC 110468). The whole genome of the strain TP-A0890 monoisolate was read by using a combined strategy of shotgun sequencing with GS FLX+ (Roche) (84 Mb of sequences, 9.5-fold coverage) and paired-end sequencing with MiSeq (Illumina) (800 Mb, 91-fold coverage). These reads were assembled using the Newbler version 2.8 software and subsequently finished using the GenoFinisher software (3), which led to a final assembly of 33 scaffold sequences >500 bp each. The total size of the assembly was 8,723,085 bp, with a G+C content of 71.6%. Coding sequences were predicted by Prodigal (4). Polyketide synthase (PKS) and nonribosomal peptide synthetase (NRPS) gene clusters were characterized in the same manner as previously described (5).

One type I PKS, one type II PKS, one type III PKS, four NRPS, and one hybrid PKS/NRPS gene clusters are present in the genome of strain TP-A0890. An NRPS (orf164) encoded in scaffold13 showed 98% sequence identity to MarM of *Streptomyces* sp. B9173. The *mar* cluster is assigned to the biosynthetic gene cluster of maremycins (6), an analogous molecule of FR-900463. Scaffold13 contained all the orthologues (orf152 to orf168) of *marA-Q*, and the orthologous proteins encoded by these genes showed >98% sequence identity to MarA-Q, respectively. This bioinformatic evidence suggests that the gene cluster in scaffold13 is responsible for FR-900452 synthesis. Type III PKS (orf150) encoded in scaffold17 showed 88 to 92% identities to NphC, NapB1, and Mcl17, which are responsible for the biosynthesis of the naphthoquinone units of furaquinocin, napyradiomycin, and merochlorin, respectively. These three compounds are polyketide-isoprenoid hybrids, and their polyketide and isoprenoid units are synthesized by type III PKS and the mevalonate pathway, respectively (7–9). The mevalonate biosynthesis genes are also contained in scaffold17 (orf131 to orf136 and orf140). Therefore, the genes contained in orf131 to orf150 were assigned to constitute the A-74863a biosynthesis gene cluster. In addition to these two clusters, six more PKS and NRPS gene clusters are present in the genome, but the sequence similarities to known genes are low, suggesting the potency of this strain to produce novel polyketides and/or nonribosomal peptides.

The genome sequence of *Streptomyces* sp. TP-A0890 revealed the presence of multiple PKS and NRPS gene clusters, which are likely silent under usual culture conditions. Further exploration of medium and/or culture conditions is in progress to activate the silent genes for the biosynthesis of unknown metabolites in this strain.

### Nucleotide sequence accession numbers.

This whole-genome sequence has been deposited in DDBJ/ENA/GenBank under the accession no. BBYG00000000. The version described in this paper is the first version, BBYG01000000.
